# Circulating tumor-associated antigen-specific IFNγ^+^4-1BB^+^ CD8^+^ T cells as peripheral biomarkers of treatment outcomes in patients with pancreatic cancer

**DOI:** 10.3389/fimmu.2024.1363568

**Published:** 2024-03-14

**Authors:** Hirotomo Murakami, Shokichi Takahama, Hirofumi Akita, Shogo Kobayashi, Yuji Masuta, Yuta Nagatsuka, Masaya Higashiguchi, Akira Tomokuni, Keiichi Yoshida, Hidenori Takahashi, Yuichiro Doki, Hidetoshi Eguchi, Nariaki Matsuura, Takuya Yamamoto

**Affiliations:** ^1^ Laboratory of Precision Immunology, Center for Intractable Diseases and ImmunoGenomics, National Institutes of Biomedical Innovation, Health and Nutrition, Osaka, Japan; ^2^ Department of Gastroenterological Surgery, Graduate School of Medicine, Osaka University, Osaka, Japan; ^3^ Department of Gastroenterological Surgery, Osaka International Cancer Institute, Osaka, Japan; ^4^ Next-Generation Precision Medicine Research Center, Osaka International Cancer Institute, Osaka, Japan; ^5^ Department of Gastroenterological Surgery, Osaka General Medical Center, Osaka, Japan; ^6^ Laboratory of Aging and Immune Regulation, Graduate School of Pharmaceutical Sciences, Osaka University, Osaka, Japan; ^7^ Department of Virology and Immunology, Graduate School of Medicine, Osaka University, Osaka, Japan

**Keywords:** pancreatic cancer, tumor-associated antigen-specific CD8 + T cells, PBMC, prognostic marker, immune checkpoint inhibition

## Abstract

CD8^+^ T cells affect the outcomes of pancreatic ductal adenocarcinoma (PDAC). Using tissue samples at pre-treatment to monitor the immune response is challenging, while blood samples are beneficial in overcoming this limitation. In this study, we measured peripheral antigen-specific CD8^+^ T cell responses against four different tumor-associated antigens (TAAs) in PDAC using flow cytometry and investigated their relationships with clinical features. We analyzed the optimal timing within the treatment course for effective immune checkpoint inhibition *in vitro*. We demonstrated that the frequency of TAA-specific IFNγ^+^4-1BB^+^ CD8^+^ T cells was correlated with a fold reduction in CA19-9 before and after neoadjuvant therapy. Moreover, patients with TAA-specific IFNγ^+^4-1BB^+^ CD8^+^ T cells after surgery exhibited a significantly improved disease-free survival. Anti-PD-1 treatment *in vitro* increased the frequency of TAA-specific IFNγ^+^4-1BB^+^ CD8^+^ T cells before neoadjuvant therapy in patients, suggesting the importance of the timing of anti-PD-1 inhibition during the treatment regimen. Our results indicate that peripheral immunophenotyping, combined with highly sensitive identification of TAA-specific responses *in vitro* as well as detailed CD8^+^ T cell subset profiling via *ex vivo* analysis, may serve as peripheral biomarkers to predict treatment outcomes and therapeutic efficacy of immunotherapy plus neoadjuvant chemotherapy.

## 1 Introduction

Pancreatic ductal adenocarcinoma (PDAC) has a poor prognosis, with a 5-year survival rate of approximately 12% (1). Resection is generally feasible in only 20% of PDAC cases (2), and the recurrence rate remains high (3). Therefore, multidisciplinary treatment with adjuvant therapies is employed to improve patient outcomes (4).

Cytotoxic chemotherapy and radiation therapy, which are commonly used for PDAC, affect immune cells, including CD8^+^ T cells. For example, gemcitabine administration increases the peripheral blood and peritumoral CD8/CD4 ratios in a mouse model of liver metastasis and peritumoral dissemination (5). Furthermore, in patients with stage III–IV PDAC, elevated exhausted peripheral blood mononuclear cell (PBMC) CD8^+^ T cells are associated with a poorer prognosis post-chemotherapy (6). Therefore, the characteristics of PBMC CD8^+^ T cells may vary depending on the pathogenesis and treatment of PDAC. However, it remains unclear whether tumor-associated antigen (TAA)-specific CD8^+^ T cells influence chemotherapy or surgery outcomes.

Recently, immunotherapy, including immune checkpoint inhibitors (ICIs), has been applied to the treatment of various cancers (7–11). However, its benefits for patients with PDAC are limited, as evidenced by the low objective response rates (3.1%) (12). The success of ICIs largely depends on the tumor microenvironment (TME); however, the use of tumor tissue biopsy samples before ICI initiation is challenging for monitoring the immunological condition of the TME. Thus, blood surrogate markers are valuable for assessing the anti-tumor efficacy of immunotherapeutic approaches. In lung cancer, correlations between the cytotoxic activity of tumor-infiltrating lymphocytes (TILs) and that of peripheral blood mononuclear cell (PBMC)-derived T cells have been reported, as have correlations between the cytotoxic activity of TILs and the percentage of PBMC-derived effector memory re-expressing CD45RA CD8^+^ T cells. These findings suggest that certain phenotypes of peripheral blood CD8^+^ T cells may reflect those of TILs (13).

In the present study, we examined the potential utility of antigen-specific CD8^+^ T cell responses against TAAs detected in the blood of patients with PDAC. By analyzing PBMCs, we investigated the relationship between TAA-specific CD8^+^ T cell responses and cytotoxic chemotherapy in patients with PDAC. Additionally, we evaluated the efficacy of immunotherapy by monitoring the TAA-specific responses of CD8^+^ T cells. We also analyzed immune response enhancement by ICI *in vitro* to determine the optimal timing of treatment to maximize the efficacy of neoadjuvant chemotherapy.

## 2 Materials and methods

### 2.1 Patient and sample collection

Patients with pancreatic cancer were recruited from the Osaka University Hospital (Osaka, Japan) and Osaka International Cancer Institute (Osaka, Japan) from September 2019 to December 2021. All patients received neoadjuvant chemotherapy or chemoradiotherapy with the following regimens: gemcitabine + nab-paclitaxcel (n=9), gemcitabine + nab-paclitaxcel + radiation (n=7), gemcitabine + S-1 (n=20), gemcitabine + S-1 + radiation (n=13), FOLFIRINOX (n=1), gemcitabine + radiation (n=1), and multiple regimens (n=6). This study was approved by the local institutional ethics committees of Osaka University, Osaka International Cancer Center, and the National Institutes of Biomedical Innovation, Health, and Nutrition, Osaka, Japan, and was conducted in accordance with the Declaration of Helsinki (1975). All participants provided written informed consent. PBMCs were isolated from patients with PDAC within 6 h of blood sampling using BD Vacutainer CPT (BD Biosciences, Franklin Lakes, NJ, USA). PBMCs were cryopreserved in fetal bovine serum (FBS) containing 10% dimethyl sulfoxide (DMSO) and stored in liquid nitrogen vapor until analysis.

### 2.2 Pancreatic cancer cell lines, quantitative reverse transcription polymerase chain reaction, and cancer cell line encyclopedia analysis

Pancreatic cancer cell lines were cultured in an appropriate culture medium supplemented with 10% FBS and 1% penicillin–streptomycin (Gibco, Thermo Fisher Scientific, Waltham, MA, USA). Dulbecco’s modified Eagle medium (Sigma-Aldrich, St. Louis, MO, USA) was used for BxPC3, MiaPaCa2, Panc1, and PSN1 cells, and Eagle’s minimal essential medium (Sigma-Aldrich) was used for SUIT-2 cells. TYPK1 cells were cultured in a 1:1 mixture of DMEM and Ham’s F-12 medium supplemented with 5% FBS and 1% penicillin–streptomycin. Cells were cultured at 37°C and 5% CO_2_ until reaching 80% confluency, at which point mRNA was extracted using an RNeasy mini kit (Qiagen, Hilden, Germany).

qRT-PCR was performed using SuperScript III Master Mix (Invitrogen, Thermo Fisher Scientific), RT enzyme, ROX, and TaqMan probes for candidate genes (*CEACAM5*, Hs00944025_m1; *CTAG1*, Hs00265824_m1; *DCT*, Hs01098278_m1; *MUC1*, Hs00159357_m1; *Telomerase reverse transcriptase (TERT)*, Hs00972656_m1; and *WT1*, Hs00240913_m1). The following thermal cycling conditions were used: 30 min at 45°C, 5 min at 95°C, 50 cycles of 1 s at 95°C, and 60 s at 50°C. qRT-PCR was replicated three times.

The CCLE (14) was used to evaluate the mRNA expression of six TAAs (*CEACAM5*, *NY-ESO-1* [*CTAG1A*], *TRP2* [*DCT*], *MUC1*, *TERT*, and *WT1*) in the pancreatic cancer cell lines. The normalized transcripts per kilobase million (TPM) dataset were downloaded from the Cancer Dependency Portal (DepMap) on 2022.12.14. The original log2 (TPM+1) values were plotted as a heatmap without scaling.

### 2.3 Overlapping peptides

Overlapping peptides covering four TAAs (all from JPT Peptide Technologies, Berlin, Germany) were used in this study: PepMix™ Human CEA (#PM-CEA) for CEACAM5; PepMix™ Human Mutin-1 (#PM-MUC1) for MUC1; PepMix™ Human TERT (#PM-TERT) for TERT; and PepMix™ Human WT1/WT33 (#PM-WT1) for WT1. The peptides consisted of 15 amino acids spanning the complete amino acid sequence of the indicated protein antigen, with 11 overlapping amino acids between adjacent peptides (**Supplementary Table 1**).

### 2.4 Amplification of TAA-specific CD8^+^ T cells by *in vitro* culture

TAA-specific CD8^+^ T cells were enriched from the PBMCs of patients with PDAC. Briefly, frozen PBMCs were thawed and treated with 1 mL of 50 unit/mL of benzonase (Merck, Rahway, NJ, USA) in R10 medium for 2 h at 37°C in 5% CO_2_. Subsequently, 20% of the PBMCs (1.1×10^5^ [0.2×10^5^ – 3.5 ×10^5^] cells) were pulsed with 2 μg/mL of each of the overlapping TAA peptides (CEA, MUC1, TERT, and WT1) for 1 h at 37°C. After washing, the pulsed PBMCs were co-cultured with the remaining 80% un-pulsed PBMCs in R10 in the presence of 20 U/mL of recombinant interleukin (IL)-2 (R&D Systems, Minneapolis, MN, USA) for 10 days; the medium was changed on days 4 and 7. For the ICI experiments, 1 mg/mL of anti-PD-1 (Cat# 621604, RRID: AB_2820105; BioLegend, San Diego, CA, USA), anti-TIGIT (Cat# 16-9500-82, RRID: AB_10718831; Thermo Fisher Scientific), or mouse IgG1 isotype control antibody (Cat# 400102, RRID: AB_2891079; BioLegend) was added to the culture medium at the beginning of the culture, and half of the medium was replaced with a culture medium without antibodies on days 4 and 7.

### 2.5 Flow cytometric detection of TAA-specific CD8^+^ T cell responses

Ten days after culture, amplified cells were stimulated with 2 μg/mL of the TAA-derived overlapping peptides (CEA, MUC1, TERT, and WT1) for 30 min at 37°C with CD107A antibody (BD Biosciences). After 30 min of stimulation, 1 μL/mL BD GolgiPlug (containing Brefeldin A) (BD Biosciences) and 0.7 μL/mL of BD GolgiStop (containing Monensin) (BD Biosciences) were added to the media, and the cells were cultured for another 5.5 h. After incubation, the cells were washed with phosphate-buffered saline (PBS) and stained using a Live/Dead Fixable Aqua Dead Cell Stain Kit (L34957; Thermo Fisher Scientific) at 18-25°C for 5 min. Subsequently, the samples were probed with antibodies (**Supplementary Table 2A**) against cell surface markers for 15 min at 18-25°C. After washing, the cells were thoroughly resuspended in 200 µL BD Cytofix/Cytoperm solution per well and incubated for 20 min at 18-25°C. The cells were washed twice with BD Perm/Wash buffer and probed with intracellular staining antibodies for 25 min at 18-25°C. After staining, the cells were washed twice with BD Perm/Wash buffer and fixed with 1% paraformaldehyde. The data were collected using FACSymphony A5 (BD Biosciences).

### 2.6 HLA typing and analysis

Cryopreserved PBMCs were thawed, and genomic DNA was extracted from a portion of cells (approximately 100,000 cells) using a QIAamp DNA mini kit (Qiagen) and stored at −30°C until use. Isolated genomic DNA was used as a template to prepare cDNA libraries for HLA typing using the commercially available kit AlloSeq™ Tx 17.1 (CareDx) or WAKFlow® HLA DNA Typing (Wakunaga Pharmaceutical Co. Ltd., Osaka, Japan). The combination of each HLA was visualized using the “*circlize*” package (version 0.4.15) in the R software language (version 4.2.1). The frequency of HLA-A types among the donors was calculated based on the cumulative total number of alleles.

### 2.7 *Ex vivo* flow cytometry profiling of bulk CD8^+^ T cells

Cryopreserved PBMCs were thawed, washed with PBS, and stained with Fixable Viable Stain UV440 (BD Biosciences) at 18-25°C for 5 min. CC-chemokine receptor 7 (CCR7) was stained at 37°C for 10 min and probed using antibodies against the remaining markers (**Supplementary Table 2B**) at 18-25°C for 15 min. After washing, the cells were thoroughly resuspended in 500 µL Fix/Perm solution per tube and incubated for 40 min at 4°C. The cells were washed twice with BD Perm/Wash solution and probed with anti-Ki67 antibodies for 40 min at 4°C. After staining, the cells were washed twice with BD Perm/Wash solution and fixed with 1% paraformaldehyde, and the data were collected using FACSymphony A5 (BD Biosciences).

### 2.8 Flow cytometry data analysis

Flow cytometry FCS files were analyzed using FlowJo software (version 10.8.1; RRID: SCR_008520; BD Biosciences). The gating setting for settings for each population are described in result section and Figures. TAA-specific CD8^+^ T cell responses were determined by subtracting the value obtained by peptide-free stimulation (DMSO; background) from that obtained by TAA stimulation. After background subtraction, values less than 0.01% were considered negative (no response). For the IFNγ^+^4-1BB^+^ criteria, values greater than 0.01% after background subtraction corresponded with responders, while those with values less than 0.01% corresponded with non-responders. For the IFNγ^+^ and/or 4-1BB^+^ criteria, if the sum of the values after the background subtraction of IFNγ^+^4-1BB^+^, IFNγ^+^4-1BB^-^, and IFNγ^-^4-1BB^+^ was greater than 0.03%, it corresponded with a responder, while if it was less than 0.03%, it corresponded with a non-responder. For the IFNγ^+^ and 4-1BB^+^ criteria, if the sum of the values after the background subtraction of two gates in each marker positive cells was greater than 0.02%, it corresponded with a responder, while if it was less than 0.02%, it corresponded with a non-responder.

### 2.9 Statistical analyses

Statistical analyses were performed using R/Bioconductor (R version 4.2.1) or GraphPad Prism (version 9.0.0; GraphPad Software, RRID: SCR_002798). Experiments and data analysis were performed by individuals blinded to the collection of blood samples and clinical information. For the HLA analysis, statistical significance of all combinations of four gating sets in each HLA type were obtained using Fisher’s exact test, and are displayed in tile format.

## 3 Results

### 3.1 Selection of specific TAAs in pancreatic cancer cells

To detect major TAA-specific PBMC CD8^+^ T cell responses in patients with PDAC, we selected TAAs based on their expression in PDAC cells. Of the 403 TAAs registered in the TAA database (TANTIGEN 2.0) (15), we selected four candidates that have been utilized in peptide vaccine clinical trials: CEACAM5 [carcinoembryonic antigen (CEA)] (16), MUC1 (17), TERT (18), and WT1 (19). Additionally, we selected two candidates used in peptide vaccine clinical trials for other cancers, NY-ESO-1 (CTAG1A) and TRP2 (DCT).

**Figure 1:**
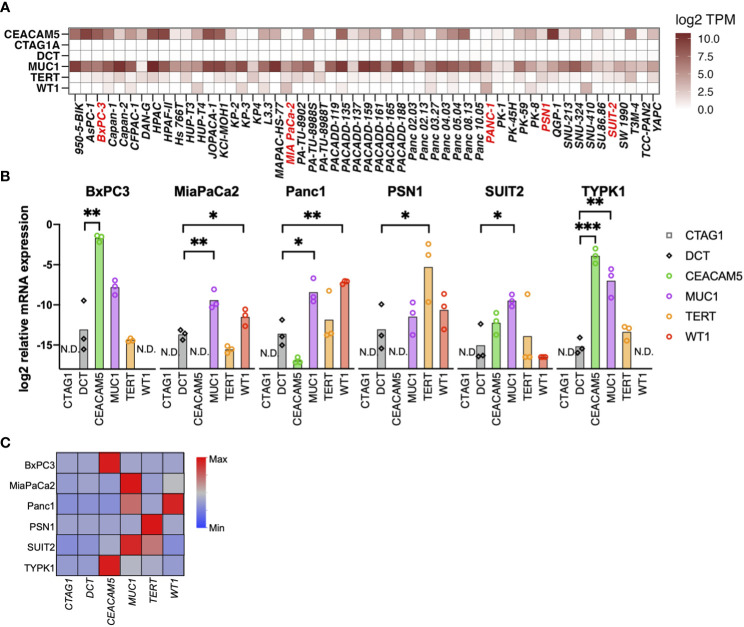
Selection of tumor-associated antigens (TAAs) specifically expressed in pancreatic cancer. **(A)** Gene expression of six TAAs (CEACAM5, NY-ESO-I [CTAG1A], TRP2 [DCT], MUC1, TERT, and WT1) in pancreatic cancer cell lines. A public database (CCLE) was used to evaluate mRNA expression using RNA-sequencing. **(B, C)** mRNA expression of TAAs in pancreatic cancer cell lines determined by qRT-PCR. Relative expression levels of the housekeeping gene (hGUS) are shown as bar graphs **(B)**, and median values after min-max normalization are shown as heatmap **(C)**. Unpaired t-test was used for statistical analyses. *P<0.05, **P<0.01, ***P<0.001. “N.D” means “Not Detected”.

Using the RNAseq data of 1404 cell lines from the CCLE, we assessed the mRNA expression of six TAAs. Of these, *CEACAM5*, *MUC1*, *TERT*, and *WT1* were expressed in multiple PDAC cell lines, whereas *CTAG1A* and *DCT* were not expressed (**Figure 1A** and **Supplementary Figure 1**). Moreover, mRNA expression was further confirmed through qRT-PCR in six PDAC cell lines (four from primary tumors, one from liver metastasis, and one from lymph node metastasis), revealing upregulation of the expression of *CEACAM5*, *MUC1*, *TERT*, and *WT1* in several cell lines (**Figures 1B, C**).

### 3.2 Establishment of a detection system for TAA-specific PBMC CD8^+^ T cell response from patients with PDAC

**Figure 2:**
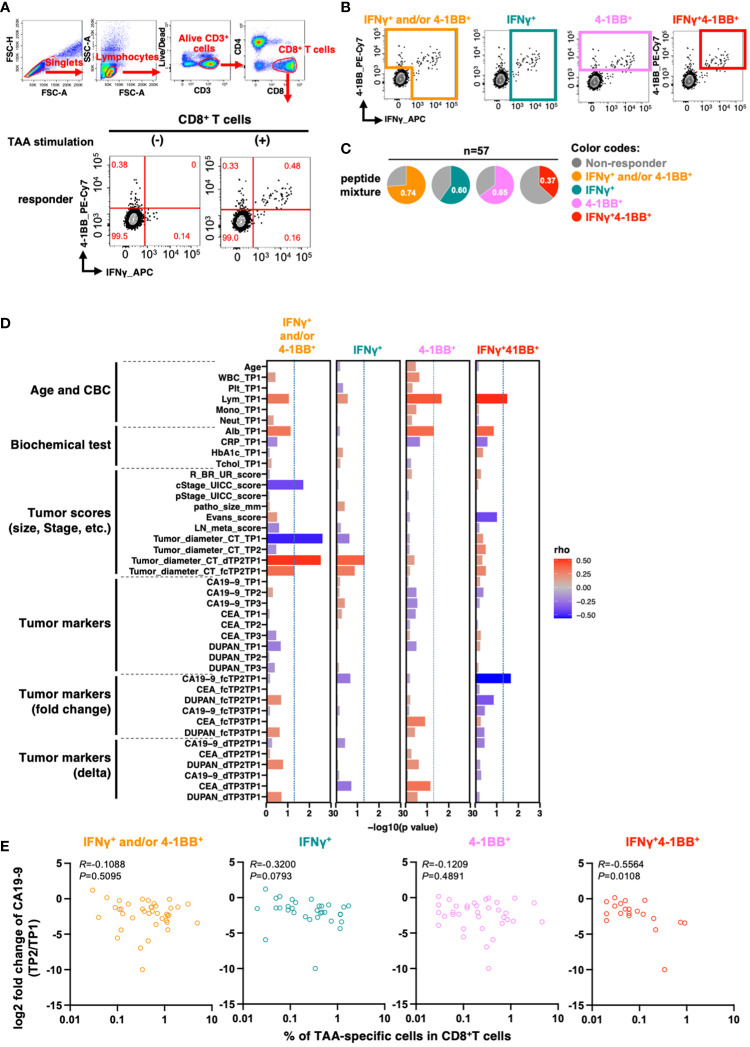
TAA-specific responses in CD8^+^ T cells in PBMCs at the beginning of treatment are associated with changes in serum CA19-9 levels before and after treatment. **(A)** Flow cytometry gating for TAA-specific CD8^+^ T cell response analysis and antigen-specific responses in CD8^+^ T cells upon stimulation of PBMCs from patients with pancreatic cancer with the mixture of TAA peptides pool. Examples of TAA-specific response-positive specimens are shown. Numbers in the gates shown in red indicate frequencies (%). **(B)** Regions considered to have TAA-specific CD8^+^ T-cell responses according to the four criteria are shown. **(C)** Percentage of patients with or without TAA-specific responses according to the four criteria (n=57). **(D)** Correlation between the percentage of TAAs-specific response in antigen-specific response-positive specimens according to the four criteria and the clinical information of the patients. The percentage of cells positive for each marker in CD8^+^ T cells without peptide stimulation (as background) was subtracted from the percentage of each marker positive cells in CD8^+^ T cells with TAA stimulation. **(E)** Correlation between the percentage of TAA-specific response in CD8^+^ T cells and changes in pre- and post-treatment serum CA19-9 levels. Patients with CA19-9 levels below the detection sensitivity were excluded. Spearman's rank correlation test was used for statistical analysis.

We then detected TAA-specific CD8^+^ T cell responses in PBMCs derived from patients with PDAC at treatment initiation. As these cells are known to have a low frequency (20), to increase detection sensitivity, we stimulated PBMCs with overlapping peptides covering the full length of the four TAAs (**Supplementary Table 1**) and then cultured them in the presence of IL-2 for 10 days to amplify TAA-specific cells (21). Moreover, we aimed to increase detection sensitivity by stimulation with a mixture of four different TAAs. After culture, we re-stimulated the cells with the peptide pool and detected TAA-specific CD8^+^ T cell responses using a flow cytometer. To evaluate antigen-specific responses, we measured the expression levels of interferon-gamma (IFNγ), a widely used marker for antigen-specific CD8^+^ T cell responses, and 4-1BB, which is known as an activation-induced marker. IFNγ and 4-1BB have each been used as markers of antigen-specific responses (22, 23), and the antigen-specific response of the two in combination has been evaluated (24). However, it was unclear whether the and/or case, single positive, or double positive is more useful in controlling cancer, so we compared them in this study based on four criteria: IFNγ^+^ and/or 4-1BB^+^, IFNγ^+^, 4-1BB^+^, and double-positive (IFNγ^+^4-1BB^+^) (**Figures 2A, B**). Although there was a certain amount of bulk T cell amplification due to culture in the presence of IL-2, there was no increase in TAA-specific CD8^+^ T cell responses due to TAA stimulation in healthy donors (**Supplementary Figure 2**).

### 3.3 Association of TAA-specific PBMC IFNγ^+^4-1BB^+^ CD8^+^ T cell responses with neoadjuvant therapy efficacy in patients with PDAC

To investigate the relationship between TAA-specific CD8^+^ T cell responses and clinical features of patients with PDAC (n=57), we stimulated PBMCs with a peptide pool containing four TAAs and determined the percentage of responders for TAA-specific CD8^+^ T cell response using the four criteria described above (**Figure 2C** and **Supplementary Figure 3**).

We subsequently investigated if these cell responses were affected by specific HLA class 1 types. Among the 57 donors, 12 HLA-A types were detected with 25 allelic combinations (**Supplementary Figures 4A, B**), and TAA-specific CD8^+^ T cell responses were observed for all HLA-A types except A*30:01 (**Supplementary Figure 4C**). We then compared the HLA distribution of responders in each criterion to that of all donors to determine whether TAA-specific CD8^+^ T cell responses were more prevalent in specific HLA-A types. However, we detected no differences in our cohort (**Supplementary Figures 4D, E**). Larger cohorts should be employed to investigate the association between HLA and TAA-specific responses.

We investigated the correlation between TAA-specific CD8^+^ T cell responses and patient characteristics at the initiation of neoadjuvant therapy. The analysis revealed a significant association between TAA-specific IFNγ^+^4-1BB^+^ CD8^+^ T cell response and pancreatic head and pancreatic body tail cancer (*p* = 0.0307; **Supplementary Table 3**). However, no significant correlations were observed for basic patient characteristics, such as age, sex, blood counts, and tumor factors (**Supplementary Table 3**). Moreover, there were no significant differences in the presence or absence of TAA-specific responses by treatment content (**Supplementary Table 3**).

To more comprehensively assess the relationship between clinical background information and total TAA-specific CD8^+^ T cell responses, we generated a new index that may reflect the effects of neoadjuvant therapy. The index comprised ratios and differences of three tumor markers (CA19-9, CEA, and DUPAN-2), along with the tumor diameter on computed tomography (CT) images at the initiation of treatment, after neoadjuvant therapy, and after surgery. We then analyzed the parameters that were correlated with the frequency of TAA-specific CD8^+^ T cell responses based on the four criteria.

The number of lymphocytes in the peripheral blood was positively correlated with TAA-specific 4-1BB^+^ CD8^+^ T cell and TAA-specific IFNγ^+^4-1BB^+^CD8^+^ T cell frequencies (**Figure 2D**, columns 3 and 4). TAA-specific CD8^+^ T cell frequency, characterized by IFNγ^+^ and/or 4-1BB^+^, was inversely correlated with both clinical progression (cStage) and tumor diameter on CT before treatment initiation. However, no inverse correlation was observed between the pathological progression (pStage) of the resected specimen and the difference in tumor diameter on CT after neoadjuvant therapy (**Figure 2D**, column 1). In contrast, the frequency of TAA-specific IFNγ^+^4-1BB^+^ CD8^+^ T cell responses showed an inverse correlation with the rate of CA19-9 change during neoadjuvant therapy: the lower the rate, the better the therapeutic effect (R = −0.56, *p* = 0.011; **Figure 2D**, column 4 and **Figure 2E**). CA19-9 is a widely used serum biomarker in PDAC, and changes in its levels during neoadjuvant therapy are considered to be prognostic (25). The results suggest that patients with TAA-specific IFNγ^+^4-1BB^+^ CD8^+^ T cell response observed prior to treatment initiation had a higher response to neoadjuvant therapy. Moreover, this criterion resulted in the lowest background; therefore, we used it for subsequent analyses.

### 3.4 Postoperative TAA-specific IFNγ^+^4-1BB^+^ CD8+ T cells and prognosis

**Figure 3:**
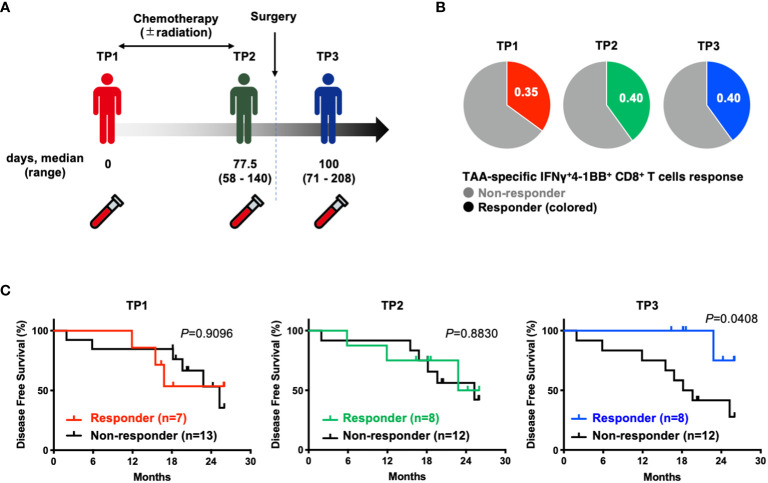
Patients with TAA-specific IFNγ+4-1BB^+^ CD8^+^ T cells responses in peripheral blood after surgery (TP3) had better disease free survival. **(A)** Schematic showing the timing of specimen collection. The numbers below each time point indicate the duration between each time point (days post TP1 at which TP2 or TP3 were sampled, median and range are indicated). **(B)** Ratio of responders evaluated by TAAs-specific IFNγ^+^4-1 BB^+^ CD8^+^ T cells at TP1/TP2/TP3. The number of donors with all time points is n=20. **(C)** Kaplan-Meier survival curves compared disease free survival between the presence or absence of TAA-specific IFNγ^+^4-1 BB^+^ CD8^+^ T cells at TP1/TP2/TP3. TP1, before the start of treatment; TP2, before surgical resection; TP3, after resection.

To investigate the prognostic impact of TAA-specific IFNγ^+^4-1BB^+^ CD8^+^ T cells at different time points (before treatment [TP1], after neoadjuvant therapy but before resection [TP2], and after resection [TP3]) using PBMCs collected from the same patients (**Figure 3A**), we compared the postoperative disease-free survival in the two groups according to the presence or absence of TAA-specific IFNγ^+^4-1BB^+^ CD8^+^ T cells. There was no difference in the number of responders at each time point (**Figure 3B**). No difference was observed before treatment [TP1] or before surgery [TP2], whereas a significant difference was observed after surgery [TP3] (**Figure 3C**). The modulation of TAA-specific IFNγ^+^4-1BB^+^ responses over time did not exhibit a consistent trend, with some patients exhibiting a loss of response and others becoming new responders before and after treatment (**Supplementary Figure 4**). Notably, the four patients who had responded before treatment and maintained this response after surgery remained recurrence-free, whereas the three patients exhibiting loss of TAA-specific IFNγ^+^4-1BB^+^ responses experienced recurrence (data not shown). There were no significant differences in clinicopathologic factors (including treatment regimen) between the presence or absence of TAA-specific IFNγ^+^4-1BB^+^ reactions in TP3 (**Supplementary Table 4**).

### 3.5 ICIs enhance TAA-specific PBMC IFNγ^+^4-1BB^+^ CD8^+^ T cell responses in cells derived from patients with PDAC

**Figure 4:**
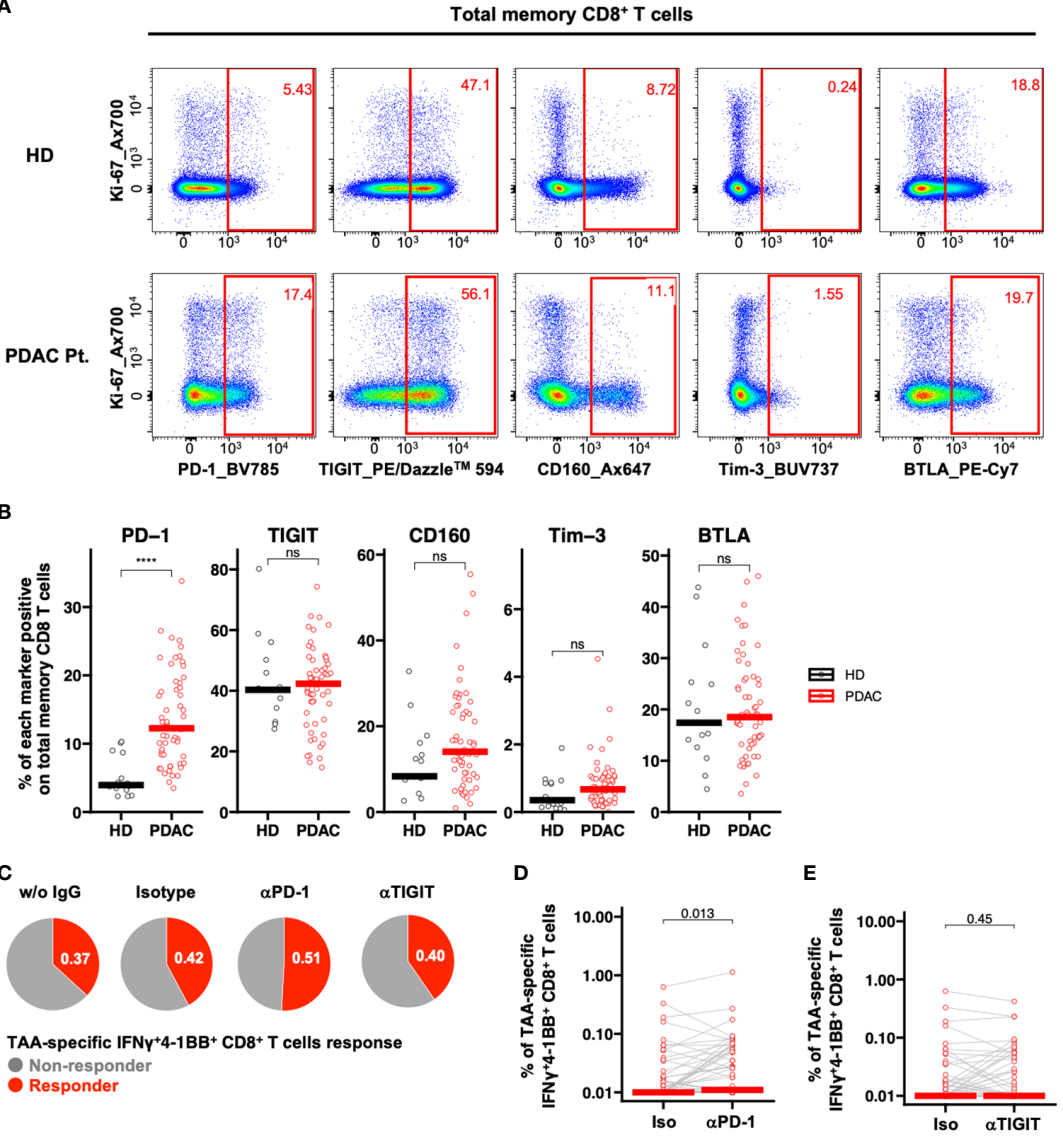
PD-1-positive memory CD8^+^ T cells are increased in PBMCs from patients with pancreatic cancer, and PD-1 inhibition *in vitro* increased TAA-specific IFNγ^+^4-1BB^+^ CD8^+^ T cells. **(A)** Representative flow cytometry plots of exhaustion markers expression in PBMCs from the healthy donor and the patient with pancreatic cancer. **(B)** PBMCs were analyzed *ex vivo* via flow cytometry, and the frequency of expression of five immune checkpoint molecules in memory CD8^+^ T cells was compared between healthy donors (HD) (n=15) and pre-treatment (TP1) pancreatic cancer patients (PDAC Pt.) (n=57). Mann—Whitney U test was used for the statistical analysis. **(C)** Ratio of responders evaluated by TAA-specific IFNγ^+^4-1BB^+^ CD8^+^ T cells in TP1 samples (n=57) treated without antibody, with isotype antibody, with anti-PD-1 antibody, or with anti-TIGIT antibody. **(D)** Comparison of TAA-specific IFNγ^+^4-1BB^+^ CD8^+^ T cells frequencies in PDAC Pt. TP1 samples (n=57) upon isotype and anti-PD-1 antibody treatment. Wilcoxon signed-rank test was used for statistical analysis. **(E)** Comparison of TAA-specific IFNy^+^4-1BB^+^ CD8^+^ T cells frequencies in TP1 samples (n=57) treated with isotype or anti-TIGIT antibody. Wilcoxon signed-rank test was used for statistical analysis. ****P<0.0001. “ns” means “not significant”.

We investigated the impact of ICIs on TAA-specific IFNγ^+^4-1BB^+^ CD8^+^ T cell response. To identify candidate immune checkpoint molecules as potential therapeutic targets, we compared the *ex vivo* expression profiles of PD-1, TIGIT, Tim-3, CD160, and BTLA on CD8^+^ Tm cells in PBMCs derived from patients with PDAC via flow cytometry (**Figure 4A**; **Supplementary Figure 6**). Among these, only PD-1 was significantly more expressed in samples derived from patients with PDAC than in those from healthy participants (**Figure 4B**). Therefore, we selected PD-1 as the target molecule. We stimulated PBMCs with TAA peptide pools in the presence of ICIs and cultured them to analyze TAA-specific IFNγ^+^4-1BB^+^ CD8^+^ T cells. We identified more responders in the anti-PD-1 antibody group than in the no-antibody and isotype groups, although this observation was not significant (**Figure 4C**; **Supplementary Figure 7**), suggesting that anti-PD-1 treatment detected new TAA-specific IFNγ^+^4-1BB^+^ CD8^+^ T cells in some non-responders. Furthermore, anti-PD-1, but not anti-TIGIT, treatment significantly increased TAA-specific IFNγ^+^4-1BB^+^ CD8^+^ T cell frequency (**Figures 4C–E**).

### 3.6 Timing of ICI intervention in PBMCs from patients with pancreatic cancer

**Figure 5:**
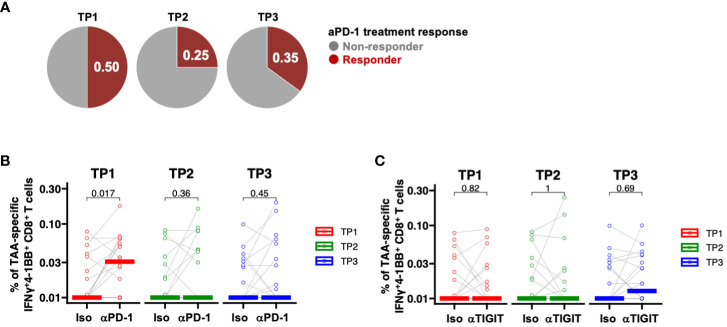
PD-1-positive memory CD8^+^ T cells are increased in PBMCs from patients with pancreatic cancer, and PD-1 inhibition *in vitro* increased TAA-specific IFNγ^+^4-1 BB^+^ CD8^+^ T cells. **(A)** Comparison of the percentage (%) of TAAs-specific IFNγ^+^4-1BB^+^ CD8^+^ T cells during anti-PD-1 antibody treatment at TP1/TP2/TP3. The number of donors with all time points is n=20. **(B)** Comparison of the percentage (%) of TAA-specific IFNy^+^4-1BB^+^ CD8^+^ T cells during anti-PD-1 treatment at TP1/TP2/TP3. The number of donors with all time points is n=20. **(C)** Comparison of the percentage (%) of TAA-specific IFNy^+^4-1BB^+^ CD8^+^ T cells during anti-TIGIT treatment at TP1/TP2/TP3. The number of donors with all time points is n=20. Wilcoxon signed-rank test was used for statistical analysis. before the start of treatment; TP2, before surgical resection; TP3, after resection.

To date, clinical trials of ICIs in patients with PDAC have included patients treated with cytotoxic anticancer drugs. Thus, we next investigated whether chemotherapy, radiation therapy, or surgery would affect the efficacy of ICI in patients with PDAC. PD-1 inhibition was assessed at the TP1, TP2, and TP3 time points using PBMCs collected from the same patients. The efficacy of ICI in donors increased as follows: 50% at TP1, 25% at TP2, and 35% at TP3 (**Figure 5A**). PD-1 inhibition significantly increased the frequency of TAA-specific IFNγ^+^4-1BB^+^ CD8^+^ T cells only at TP1 (**Figure 5B**), whereas TIGIT inhibition did not result in significant changes at any time point (**Figure 5C**). These results suggest that anti-PD-1 ICIs may be less effective after chemotherapy, either alone or in combination with radiotherapy, which could explain the poor outcomes reported in previous clinical trials.

## 4 Discussion

In the present study, we demonstrated that the frequency of TAA-specific IFNγ^+^4-1BB^+^ CD8^+^ T cells in the blood before treatment was correlated with reduced CA19-9 levels, suggesting the potential utility of the proposed method for detecting TAA-specific IFNγ^+^4-1BB^+^ CD8^+^ T cells as a surrogate marker to predict treatment efficacy. Additionally, we demonstrated that postoperative IFNγ^+^4-1BB^+^ TAA-specific IFNγ^+^4-1BB^+^ CD8^+^ T cells may be predictive of postoperative recurrence. Although it remains unclear whether this peripheral *in vitro* response reflects *in vivo* suppression of cancer cells, patients exhibiting a TAA-specific IFNγ^+^4-1BB^+^ response after surgery exhibited a favorable prognosis, whereas those exhibiting a response before treatment but not after surgery were more susceptible to recurrence.

TIL analysis requires invasive tissue collection, posing challenges to monitoring changes in patients. In contrast, liquid biopsies can be conducted multiple times with minimal invasiveness, and are considered a rich source of information that reflects biological status, therapeutic response, and clinical outcomes (26). Recently, a variety of biomolecules or particles in plasma such as cell free DNA (cfDNA), circulating tumor DNA (ctDNA), non-coding RNAs, and exome or extracellular vesicles have emerged as new probes to examine the biological status of tumors, therapeutic responses, and prognoses (27–32). In addition, certain types of cells, such as circulating tumor cells (CTCs), have been used to identify tumor status (33, 34). In general, cell-based analysis requires more labor than molecular-based analysis. However, the overall information it supplies is richer. Circulating immune cells would be as valuable a source of information as CTCs. Compared to the rare nature of CTCs and the specific tools needed to capture them, circulating T cells are easier to detect and more easily accessible using standard immunological instruments. Indeed, the transcriptomic analysis of bulk peripheral CD8^+^ T cells in melanoma patients has revealed the association between peripheral CD8^+^ T cell characteristics and ICI responses (35). Furthermore, in clinical trials investigating the use of atezolizumab and personalized RNA neoantigen vaccines, as well as mFOLFIRINOX as adjuvant chemotherapy in patients with pancreatic cancer, those with vaccine antigen-specific T cells in their PBMCs exhibited a significantly improved recurrence-free survival (36). While more patients are required for further validation, our study suggests a potential synergistic effect of immune response and neoadjuvant therapy, providing important insights for future combination therapies with anticancer drugs and ICIs.

In addition to conventional treatments such as surgery, chemotherapy, and radiation, immunotherapy has emerged as a new treatment strategy for cancer. However, the effectiveness of ICI monotherapy is limited in pancreatic cancer (10]. Therefore, various combination therapies are being investigated, including cytotoxic chemotherapy and radiotherapy (37). Moreover, a previous report has suggested that neoadjuvant chemotherapy activates the immune response (38). In cancers other than pancreatic cancer, ICI treatment is more effective when administered before surgery rather than after (39–42). Notably, a preoperative cytotoxic anticancer drug plus ICI therapy showed efficacy in a phase 3 trial for resectable non-small cell lung cancer (43). Based on these reports, clinical trials investigating ICI treatment in combination with neoadjuvant chemotherapy regimens are currently underway for pancreatic cancer. The results of these trials are anticipated to provide insights into the optimal timing of treatment. However, most of the study focuses on the neoadjuvant (preoperative) versus adjuvant (postoperative) difference, rather than the untreated versus neoadjuvant in a preoperative setting (44), or by comparing it with or without ICI in the neoadjuvant setting (45). Here, we directly investigated the three distinct timings of PD-1 inhibition *in vitro*. Contrary to the anticipated result from previous studies, our results indicated the effectiveness of PD-1 inhibition in TAA-specific IFNγ^+^4-1BB^+^ CD8^+^ T cells before the start of neoadjuvant therapy, at least *in vitro*. There is one possible explanation for this discrepancy. The rationale for neoadjuvant ICI therapy is the following: increased priming of tumor-specific T cells due to immunogenic cell death in tissue that is induced by neoadjuvant increases the antigenic stimuli (46–49). In our analysis, in contrast, TAA-specific peripheral memory CD8^+^ T cells were stimulated by an abundant amount of TAA peptides either in the presence or absence of PD-1 inhibition. Another explanation is that the study design of previous reports was limited to fully delineate the optimal timing of ICI therapy. Consistently, one study focusing on the sequence of ICI before and after neoadjuvant suggested that the ICI before neoadjuvant was efficacious in BRAF-wildtype metastatic melanoma (50).

This study has some limitations. First, this study suggests the prognostic value of analyzing postoperative peripheral blood samples in patient follow-up. However, further investigations are warranted, owing to the small number of cases and short observation period. Second, given the interplay of peripheral CD8^+^ cells with tissue-resident memory CD8^+^ T cells against anti-tumoral immunity (51), TAA-specific IFNγ^+^4-1BB^+^ CD8^+^ T cells in the blood may contribute to the efficacy of neoadjuvant therapy. However, our study did not directly analyze TILs, and it remains unclear if similar cells existed in the tissues.

In summary, we selected TAA-specific antigen molecules to stimulate CD8^+^ T cells and we established a flow cytometry system to detect antigen-specific responses of these cells in peripheral blood derived from patients with PDAC, utilizing them as peripheral biomarkers for assessing the efficacy of neoadjuvant chemotherapy. Evaluation of the impact of immunotherapy by monitoring the TAA-specific IFNγ^+^4-1BB^+^ responses of CD8^+^ T cells suggested that PD-1 inhibition may effectively increase TAA-specific IFNγ^+^4-1BB^+^ CD8^+^ T cells when administered as a neoadjuvant therapy. Our findings suggest that a sequential treatment approach, involving initial ICI treatment followed by neoadjuvant chemotherapy, as opposed to a combination therapy where ICI and neoadjuvant chemotherapy are administered simultaneously, may optimize the efficacy of multidisciplinary treatment.

## Data availability statement

The raw data supporting the conclusions of this article will be made available by the authors, without undue reservation.

## Ethics statement

The studies involving humans were approved by Ethical Review Board Osaka University Hospital Osaka Prefectural Hospital Organization Osaka International Cancer Institute Certified Review Board Research Ethics Review Committee of National Institutes of Biomedical Innovation. The studies were conducted in accordance with the local legislation and institutional requirements. The participants provided their written informed consent to participate in this study.

## Author contributions

HM: Conceptualization, Data curation, Formal analysis, Investigation, Visualization, Writing – original draft. ST: Conceptualization, Formal analysis, Investigation, Methodology, Visualization, Writing – original draft. HA: Conceptualization, Funding acquisition, Investigation, Resources, Validation, Writing – original draft, Writing – review & editing. SK: Resources, Writing – review & editing. YM: Data curation, Formal analysis, Writing – review & editing. YN: Data curation, Resources, Writing – review & editing. MH: Resources, Writing – review & editing. AT: Resources, Writing – review & editing. KY: Resources, Writing – review & editing. HT: Resources, Writing – review & editing. YD: Resources, Writing – review & editing. HE: Resources, Writing – review & editing. NM: Resources, Writing – review & editing. TY: Conceptualization, Funding acquisition, Investigation, Methodology, Project administration, Writing – original draft, Writing – review & editing.
