# Supplementary material for: Circulating tumor-associated antigen-specific IFNγ+4-1BB+ CD8+ T cells as peripheral biomarkers of treatment outcomes in patients with pancreatic cancer
Source: Front Immunol. 2024 Mar 14;15:1363568. doi: 10.3389/fimmu.2024.1363568 (PMC10972947; doi:10.3389/fimmu.2024.1363568)
Supplement: Supplementary file 1 [file DataSheet_1.pdf]

Supplementary Fig. S1

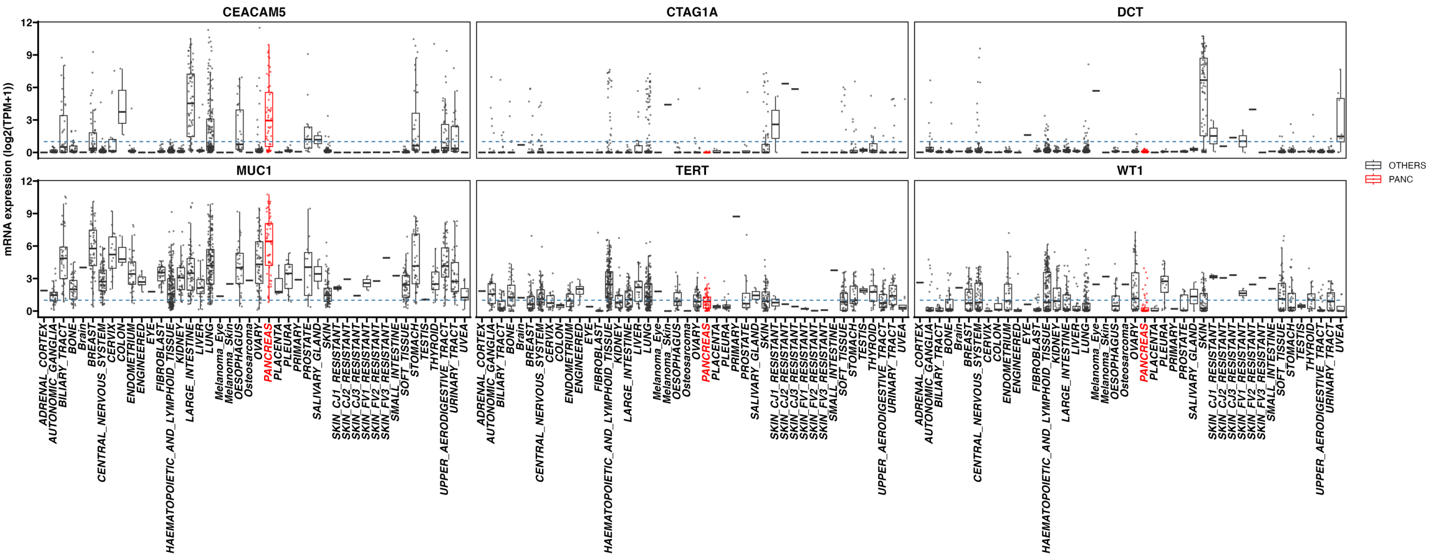

Supplementary Fig. S1. Expression of TAAs in organ-specific cancer cell lines.
mRNA expression of six TAAs (CEACAM5, NY-ESO-1 [CTAG1A], TRP2 [DCT], MUC1, TERT, and WT1) in cancer cell lines by organ obtained from a public database (CCLE). Expression in the pancreas is indicated in red. Dashed line indicates no expression (raw TPM=0). The six types of TAAs are highlighted in red.

Supplementary Fig. S2

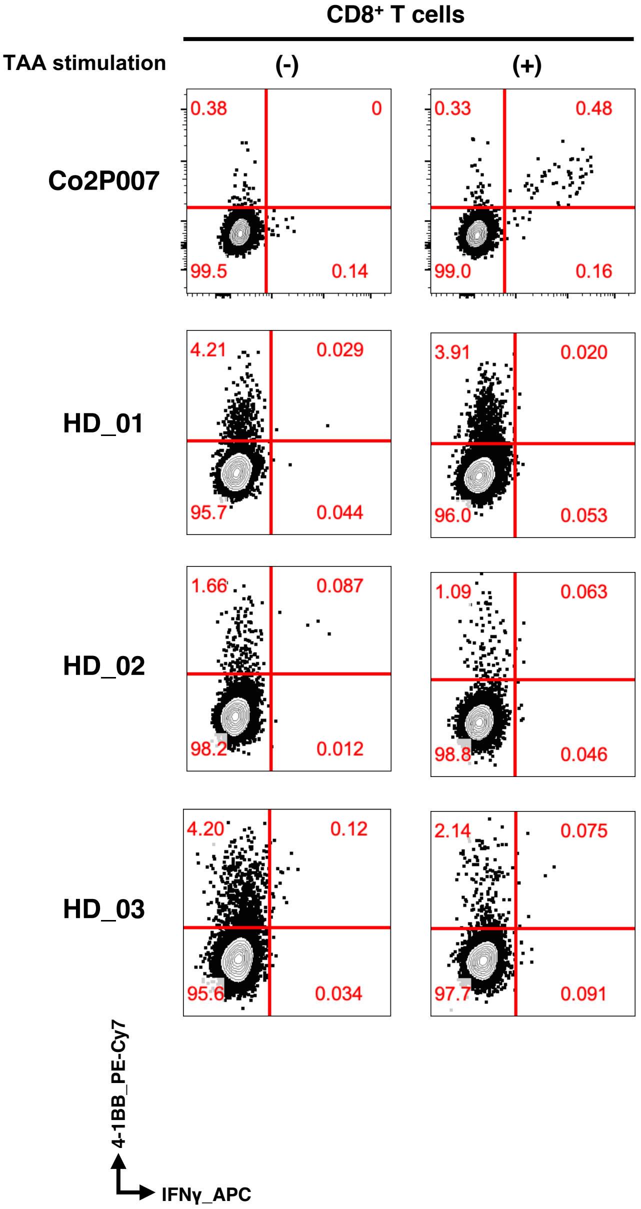

Supplementary Fig. S2. TAA-specific CD8+ T cell responses were observed in PBMCs derived from PDAC patients but not healthy donors.
Flow cytometry gating for antigen-specific responses in CD8+ T cells upon stimulation of PBMCs from patients with pancreatic cancer and healthy donor (HD, n=3) with the mixture of TAA peptides pool. Numbers in the gates shown in red indicate frequencies (%).

Supplementary Fig. S3

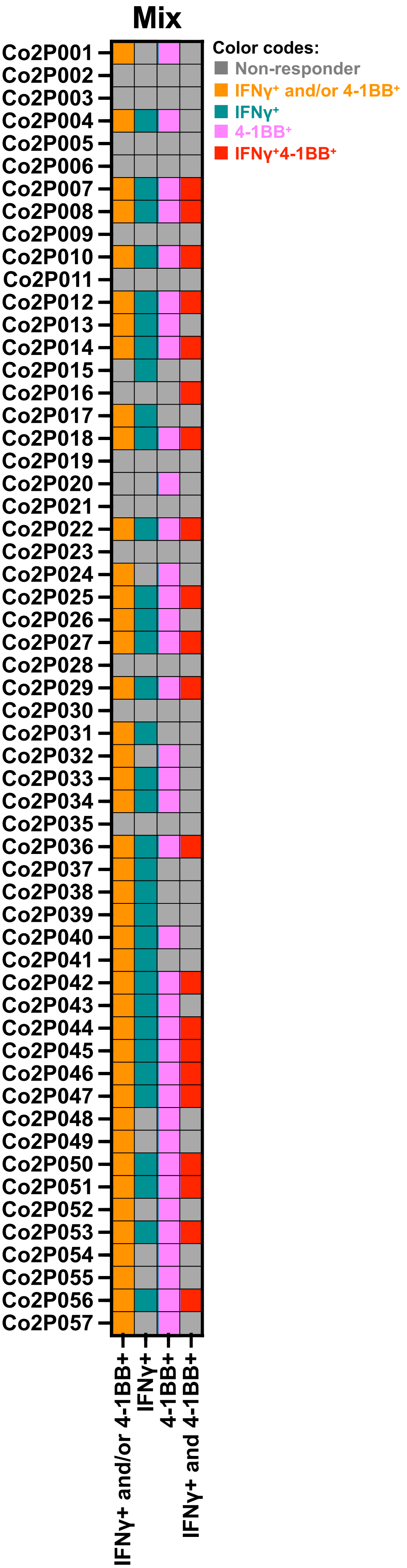

Supplementary Fig. S3. Detection of TAA-specific CD8^{+} T cells from PBMCs of patients with pancreatic cancer.
IFNγ^{+} and/or 4-1BB^{+} / IFNγ^{+} / 4-1BB^{+} / IFNγ^{+}4-1BB^{+} response of each donor in Cohort.

Supplementary Fig. S4

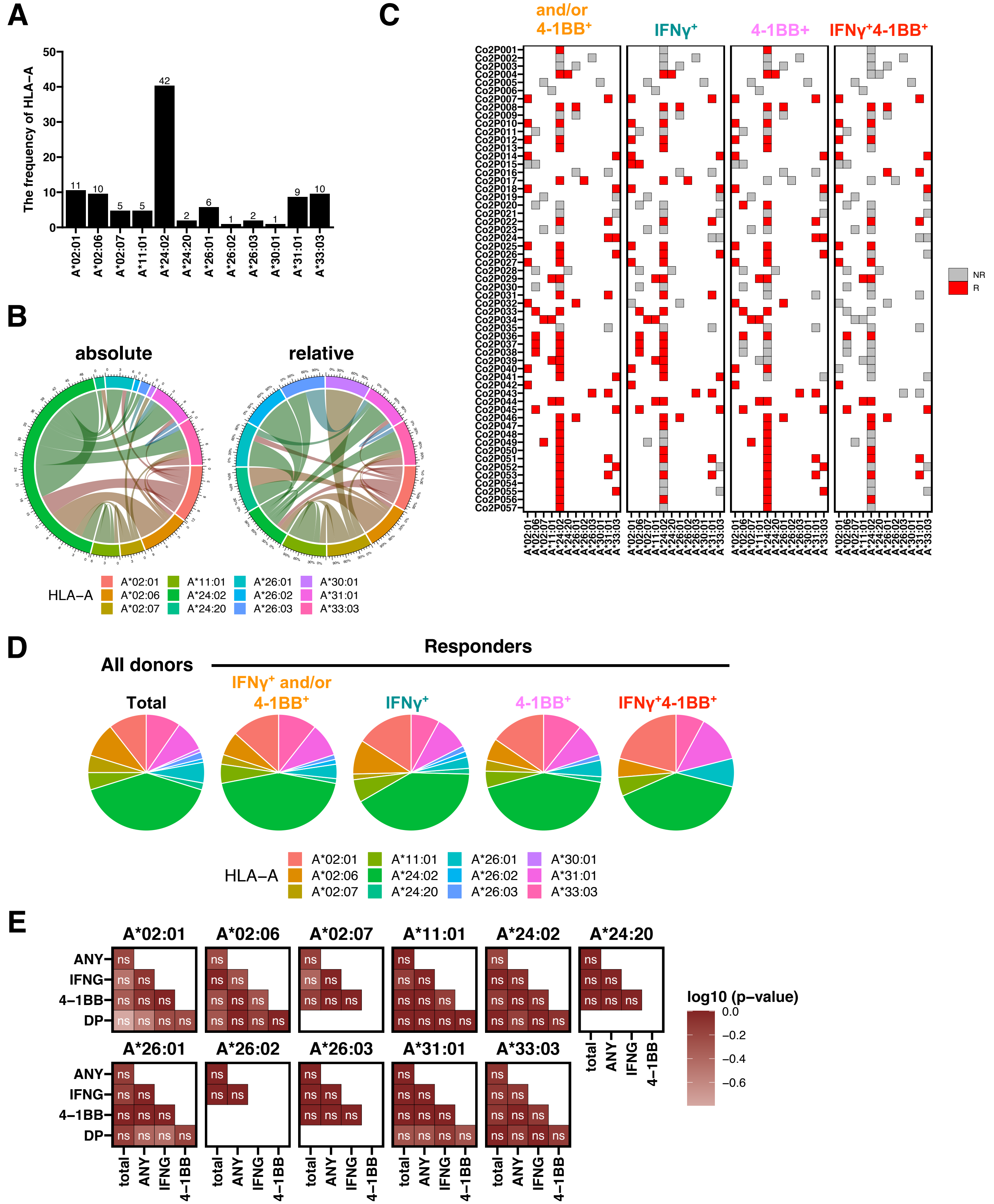

Supplementary Fig. S4. HLA-A analysis of patients with pancreatic cancer (n=57).
A, Frequency and absolute number of each HLA-A genotype (four digits) of donors (N=57) in cohort 2. Numbers above each bar indicate the number of donors. **B,** Circular visualization of each HLA-A combination. Left: plot according to the number of donors; right: plot relative to a constant number for each HLA. The color of the circular bands corresponds to each HLA-A genotype. **C,** TAA-specific responses (IFNγ+ and/or 4-1BB+, IFNγ+, 4-1BB+, and IFNγ+4-1BB+) for each HLA-A type. **D,** Pie chart of the frequency of each HLA-A among all patients and those with TAA-specific reactions. The colors correspond to each HLA-A genotype. **E,** Significant differences in the number of donors showing overall and TAA-specific responses for each HLA-A genotype evaluated using Fisher's exact probability test.

Supplementary Fig. S5

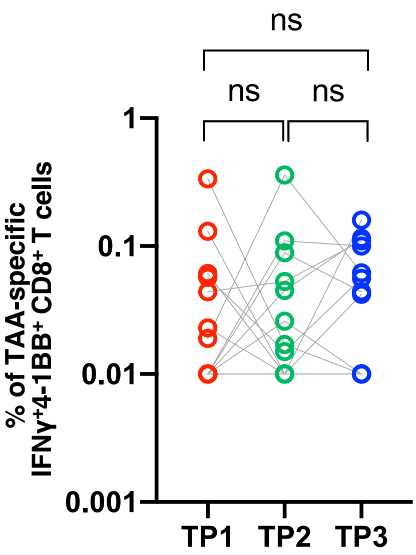

Supplementary Fig. S5. TAA-specific IFNγ^{+}4-1BB^{+} CD8^{+} T cell responses were transitioned by time points. Comparison of the percentage (%) of TAA-specific IFNγ^{+}4-1BB^{+} cells at TP1/TP2/TP3. The number of donors with all time points is n=20. Wilcoxon signed-rank test was used for statistical analysis.

Supplementary Fig. S6

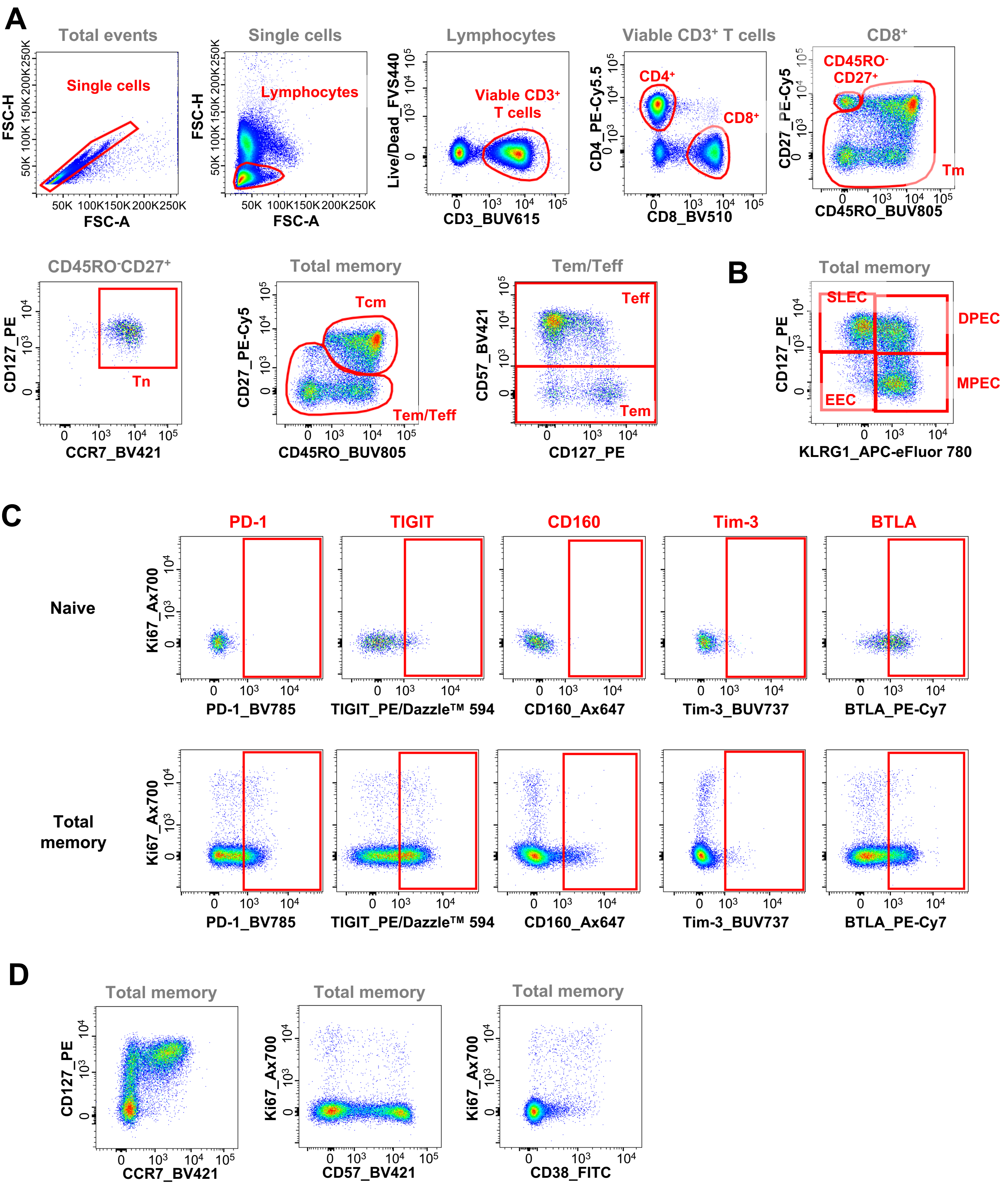

Supplementary Fig. S6. Gating diagram for the ex vivo analysis of PBMCs from patients with pancreatic cancer. Gating of naïve/memory gates and each memory subset (**A**), total memory CD8+ T cells (EEC/SLEC/MPEC/DPEC) (**B**),naïve and total memory CD8+ T cells for each checkpoint molecule (**C**), and other phenotypes of total memory CD8+ T cells (**D**) in the *ex vivo* analysis. Tn, naïve; Tm, total memory; Tcm, central memory; Tem, effector memory; Teff, effector; EEC, early effector cells; SLEC, short-lived effector cells; MPEC, memory-precursor effector cells; DPEC, double-positive effector cells.

Supplementary Fig. S7

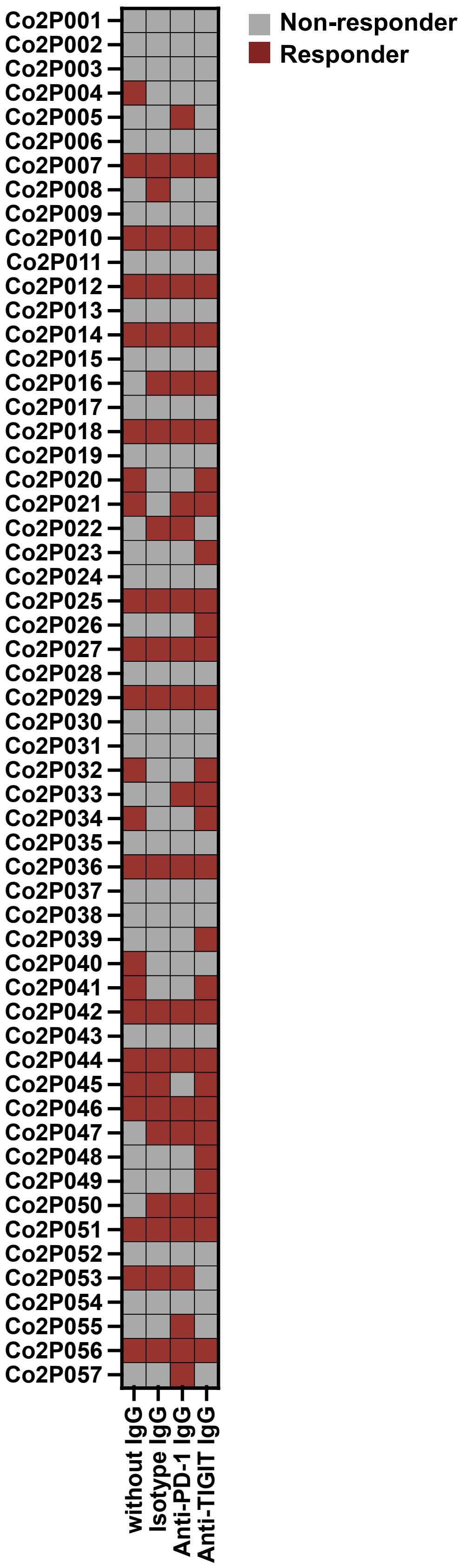

Supplementary Fig. S7 Detection of TAA-specific IFNγ^{+}4-1BB^{+} CD8^{+} T cell responses upon antibody treatment. Detection of response to no antibody treatment and treatment with isotype antibody, anti-PD-1 antibody, or anti-TIGIT antibody in each donor (n=57).
