# Supplementary material for: Circulating tumor-associated antigen-specific IFNγ+4-1BB+ CD8+ T cells as peripheral biomarkers of treatment outcomes in patients with pancreatic cancer
Source: Front Immunol. 2024 Mar 14;15:1363568. doi: 10.3389/fimmu.2024.1363568 (PMC10972947; doi:10.3389/fimmu.2024.1363568)
Supplement: Supplementary file 2 [file Table_1.pdf]

Supplementary Table S1.

	Protein Name	Design	Number of peptides	Protein Length (Amino Acid)
CEA	Carcinoembryonic antigen-related cell adhesion molecule 5	15 mers<br>11 aa overlap	173	702
hTERT	Telomerase transcriptase	15 mers<br>11 aa overlap	128	523
MUC1	Mucin-1	15 mers<br>11 aa overlap	115	1255
WT1	Wilms tumor protein	15 mers<br>11 aa overlap	110	449

Supplementary Table S2. List of TAA peptides used to detect TAA-specific $CD8^+$ T cells.

Supplementary Table S2.

A

Antigen	Clone	Fluorochrome	Company	Cat#	RRID	Staining
**CD4**	S3.5	PE-Cy5.5	Invitrogen	MHCD0418	AB_10376013	Surface
**4-1BB**	4B4-1	PE-Cy7	Biolegend	309818	AB_2207741	ICS
**IFNg**	4S.B3	APC	Biolegend	502512	AB_315237	ICS
**CD3**	SP34-2	APC-Cy7	BD Biosciences	557757	AB_396863	Surface
**Fixable Viability Stain Aqua**		BV510	Thermo Fisher Scientific	L34957		Live/Dead
**CD8**	RPA-T8	BV785	Biolegend	301046	AB_2563264	Surface

B

Antigen	Clone	Fluorochrome	Company	Cat#	RRID	Staining
**CD38**	HIT2	FITC	BD Biosciences	555459	AB_395852	Surface
**CD57**	NK-1	BB750	BD Biosciences	624391		Surface
**CD127**	A019D5	PE	Biolegend	351304	AB_10720185	Surface
**TIGIT**	A15153G	PE/Dazzle594	Biolegend	372716	AB_2632931	Surface
**CD27**	1A4CD27	PE-Cy5	Beckman Coulter	6607107		Surface
**CD4**	S3.5	PE-Cy5.5	Invitrogen	MHCD0418	AB_10376013	Surface
**BTLA**	MIH26	PE-Cy7	Biolegend	344516	AB_2629566	Surface
**CD160**	BY55	Ax647	Biolegend	341204	AB_2074410	Surface
**Ki67**	B56	Ax700	BD Biosciences	561277	AB_10611571	TF.B
**KLRG-1**	13F12F2	APC-eFluor780	Invitrogen	47-9488-42	AB_2688198	Surface
**CCR7**	G043H7	BV421	Biolegend	353208	AB_11203894	37°C
**CD8**	RPA-T8	BV510	Biolegend	301048	AB_2561942	Surface
**PD-1**	EH12-2H7	BV785	Biolegend	329930	AB_2563443	Surface
**Fixable Viability Stain 440UV**		440UV	BD Biosciences	566332	AB_2869748	Live/Dead
**CD3**	SP34-2	BUV615	BD Biosciences	751249	AB_2875266	Surface
**Tim-3**	7D3	BUV737	BD Biosciences	748820	AB_2873223	Surface
**CD45RO**	UCHL1	BUV805	BD Biosciences	748367	AB_2872786	Surface

Supplementary Table S2. FACS panel for TAA-specific CD8^{+} T cells detection (A) and *ex vivo* profiling of bulk CD8^{+} T cells (B).

Supplementary Table S3.

Parameter	total Cohort 2 (n=57)	Responder in IFNγ$^+$ and/or 4-1BB$^+$ (n=42)	P value vs non-responder	Responder in IFNγ$^+$ (n=34)	P value vs non-responder	Responder in 4-1BB$^+$ (n=37)	P value vs non-responder	Responder in IFNγ$^+$4-1BB$^+$ (n=21)	P value vs non-responder
Age (yr), median [range]	69 [41, 83]	68 [41, 83]	0.1543	67 [53, 82]	0.0633	67 [41, 83]	0.1155	65 [54, 82]	0.1018
Sex (Female/Male), n	23 / 34	17 / 25	0.9743	16 / 18	0.2094	13 / 24	0.2750	10 / 11	0.4673
Past history of cancer (no/yes), n	48 / 9	36 / 6	0.6024	29 / 5	0.7850	31 / 6	0.9043	19 / 2	0.3422
Diabetes (no/yes), n	30 / 27	21 / 21	0.5055	18 /16	0.9546	20 / 17	0.7699	10 / 11	0.6374
White Blood Cells (/µL), median [range]	5180 [2530, 9990]	5060 [2830, 9990]	0.4575	5060 [2830, 8550]	0.1668	5020 [2830, 9990]	0.4619	4860 [2830, 8100]	0.1263
Neutrophils (/µL) , median [range]	3200 [1460, 7340]	3010 [1460, 6230]	0.1421	2940 [1460, 7340]	0.0529	2890 [1460, 6230]	0.2038	2750 [1460, 6230]	0.1793
Lymphocytes (/µL) , median [range]	1140 [670, 3260]	1170 [850, 3260]	0.0955	1120 [850, 2590]	1.0000	1140 [850, 3260]	0.2418	1090 [700, 2440]	0.7833
Monocytes (/µL) , median [range]	300 [130, 600]	310 [150, 600]	0.1920	300 [150, 520]	0.6724	320 [150, 600]	0.1199	310 [130, 520]	0.3480
Platelets ($\times 10^3$/µL) , median [range]	201 [114, 389]	195 [114, 389]	0.5619	188 [114, 370]	0.1643	201 [114, 389]	0.8936	187 [114, 284]	0.2892
C-reactive protein (mg/dL) , median [range]	0.07 [<0.04, 5.57]	0.07 [<0.04, 5.57]	0.6551	0.07 [<0.04, 5.57]	0.8571	0.07 [<0.04, 5.57]	0.6802	0.06 [<0.04, 1.15]	0.3795
Albumin (g/dL) , median [range]	4.0 [3.1, 4.9]	4.0 [3.1, 4.9]	0.0203	4.0 [3.2, 4.9]	0.9870	4.0 [3.1, 4.9]	0.0284	4.0 [3.2, 4.9]	0.9094
Serum CA19-9 (U/mL) , median [range]	385 [<2, 9194]	317 [<2, 9194]	0.1982	289 [<2, 9194]	0.1988	298 [<2, 9194]	0.2697	151.5 [<2, 9194]	0.2929
Serum CEA (ng/mL) , median [range]	3 [<1, 20.5]	3 [<1, 20.5]	0.4610	2.8 [<1, 20.5]	0.5134	3 [<1, 17.2]	0.7243	2.2 [<1, 17.2]	0.2764
Serum DUPAN-2 (U/mL), median [range]	166 [<25, >4800]	140 [<25, >4800]	0.2506	149 [<25, >4800]	0.3054	149 [<25, >4800]	0.5837	170 [<25, >4800]	0.6169
cStage (I/II/III), n	46 / 4 / 7	35 / 3 / 4	0.5688	29 / 2 / 3	0.5502	31 / 3 / 3	0.4070	17 / 2 / 2	0.7764
NCCN Resectability (R / BR-PV / BR-A), n	32 / 12 / 13	25 / 8 / 9	0.6828	21 / 8 / 5	0.2075	22 / 7 / 8	0.7788	11 / 6 / 4	0.5292
Location (head / body or tail), n	37 / 20	29 / 13	0.2737	21 / 13	0.5449	25 / 12	0.5678	10 / 11	0.0307
Tumor size in CT (mm) , median [range]	21 [12, 45]	22 [12, 45]	0.7576	20 [12, 45]	0.3123	22 [12, 45]	0.5632	20 [12, 45]	0.3993
Regimen (GnP / GS / Other), n	16 / 33 / 8	13 / 24 / 5	0.6053	9 / 21 / 4	0.7392	11 / 22 / 4	0.6284	5 / 13 / 3	0.8884
Radiation (-/+), n	32 / 25	26 / 16	0.1422	22 / 12	0.1131	22 / 15	0.4922	14 / 7	0.2577
Resection (-/+), n	10 / 47	6 / 36	0.2792	6 / 28	0.9801	3 / 34	0.0108	3 / 18	0.6213

Supplementary Table S3. TAA-specific CD8$^+$ T cell responses and patients background at the start of preoperative treatment

BR-A, borderline resectable with artery involvement; BR-PV, borderline resectable with portal vein involvement; R, resectable; GnP, gemcitabine + nab-paclitaxcel; GS, gemcitabine + S-1; Other, gemcitabine or FOLFIRINOX or multiple regimen.

Supplementary Table S4.

Parameter	Responder in IFNγ^{+}4-1BB^{+} at TP3 (n=8)	Non-responder in IFNγ^{+}4-1BB^{+} at TP3 (n=12)	P value vs non-responder
Age (yr), median [range]	74 [62, 83]	67 [55, 82]	0.0893
Sex (Female/Male), n	4 / 4	2 / 10	0.1116
Location (head / body or tail), n	4 / 4	7 / 5	0.7138
NCCN Resectability (R / BR-PV / BR-A), n	6 / 2 / 0	11 / 0 / 1	0.0887
Regimen (GnP / GS / Gem), n	1 / 6 / 1	4 / 8 / 0	0.2472
Radiation (-/+), n	7 / 1	11 / 1	0.7630
pStage (0/I/II/III), n	0 / 2 / 4 / 2	1 / 5 / 2 / 4	0.7764
Tumor size (mm) , median [range]	23 [9, 55]	14 [0, 30]	0.1039
Lymph node metastasis (-/+), n	3 / 5	6 / 6	0.5808

Supplementary Table S4. TAA-specific IFNγ^{+}4-1BB^{+} CD8^{+} T cell responses and patients background after surgery

BR-A, borderline resectable with artery involvement; BR-PV, borderline resectable with portal vein involvement; R, resectable; GnP, gemcitabine + nab-paclitaxcel; GS, gemcitabine + S-1; Gem, gemcitabine.
